# Biological agents and the aging brain: glial inflammation and neurotoxic signaling

**DOI:** 10.3389/fragi.2023.1244149

**Published:** 2023-08-15

**Authors:** Amanda S. Latham, Julie A. Moreno, Charlize E. Geer

**Affiliations:** ^1^ Department of Environmental and Radiological Health Sciences, College of Veterinary Medicine and Biomedical Sciences, Colorado State University, Fort Collins, CO, United States; ^2^ Brain Research Center, Colorado State University, Fort Collins, CO, United States

**Keywords:** astrocytes, microglia, aging brain, pathogens, bacteria, viruses, inflammation, neuroinflammation

## Abstract

Neuroinflammation is a universal characteristic of brain aging and neurological disorders, irrespective of the disease state. Glial inflammation mediates this signaling, through astrocyte and microglial polarization from neuroprotective to neurotoxic phenotypes. Glial reactivity results in the loss of homeostasis, as these cells no longer provide support to neurons, in addition to the production of chronically toxic pro-inflammatory mediators. These glial changes initiate an inflammatory brain state that injures the central nervous system (CNS) over time. As the brain ages, glia are altered, including increased glial cell numbers, morphological changes, and either a pre-disposition or inability to become reactive. These alterations induce age-related neuropathologies, ultimately leading to neuronal degradation and irreversible damage associated with disorders of the aged brain, including Alzheimer’s Disease (AD) and other related diseases. While the complex interactions of these glial cells and the brain are well studied, the role additional stressors, such as infectious agents, play on age-related neuropathology has not been fully elucidated. Both biological agents in the periphery, such as bacterial infections, or in the CNS, including viral infections like SARS-CoV-2, push glia into neuroinflammatory phenotypes that can exacerbate pathology within the aging brain. These biological agents release pattern associated molecular patterns (PAMPs) that bind to pattern recognition receptors (PRRs) on glial cells, beginning an inflammatory cascade. In this review, we will summarize the evidence that biological agents induce reactive glia, which worsens age-related neuropathology.

## 1 Introduction

By 2050, populations over 65 years old are projected to double, and over 80 years expected to triple (IISD, 2023). This is due, in part, to increased availability of healthcare and disease-preventing services that have drastically improved life expectancy. With these population changes impending, it is critical that we obtain a better understanding of age-related pathologies. Age is the greatest risk factor for the formation and progression of neurological disease, including dementia, Alzheimer’s Disease (AD), Parkinson’s Disease (PD), and Lewy body dementia (Hou et al., 2019). Glial dysfunction, neuroinflammation, neuronal loss, and the accumulation of toxic protein aggregates are implicated in the aged brain. Although age-related pathologies in the brain have been researched, the role of glia in the manifestation of neurological diseases is not fully understood. In the aging brain, cellular senescence and prolonged secretion of pro-inflammatory molecules occurs. Informally described as “inflammaging,” these changes induce a unique environment where unnecessary damage to the brain is combined with an inability to perform homeostatic functions. These alterations in glial performance mimic those found in response to infectious agents, which push glia into a state of activation and make them more sensitive to subsequent stimuli. This is pertinent due to not only the increased susceptibility to infection that occurs with age, but the life-long history of accumulated illness in a person’s lifetime. In this review, we discuss how the dynamic behavior of glia is influenced by age, and how infection with various biological agents could potentially exacerbate underlying pathology.

## 2 Microglia perform neuroprotective and neurotoxic functions in the healthy brain

Microglia are the resident immune cells of the brain, derived from embryonic yolk sac precursors, similar in fashion to the resident macrophages of other tissues (Gomez Perdiguero et al., 2015). Their ability to self-renew establishes them as a unique population within the central nervous system (CNS). These cells have the longest lifespan of any documented immune cell, with populations surviving as long as 15 months in murine models (Füger et al., 2017). As their status as resident macrophage establishes, they execute immune functions in the brain, including immunological surveillance that results in the detection and elimination of pathogens. That is why microglia express major histocompatibility complex II (MHCII) receptors like other professional antigen-presenting immune cells (Hayes et al., 1987). Alongside this role, microglia maintain homeostasis within the CNS. Their phagocytic ability allows them to support neurons, by performing synaptic pruning, and they stimulate oligodendrocyte progenitor cells for proper myelinogenesis (Hagemeyer et al., 2017). Under pathologic conditions, microglia activate into neuroprotective and neurotoxic phenotypes, depending on the stimulus.

Neuroprotective microglia are produced in response to inflammation and damage, promoting neurogenesis, phagocytosing debris, and reducing inflammatory signaling in the brain (Chen and Trapp, 2016). However, during cellular stress microglia can polarize into an ameboid, neurotoxic phenotype commonly associated with chronic disease states and brain damage, which is why they are often called disease-associated microglia (DAM) (Stence et al., 2001). Binding of their toll-like receptors (TLRs) and pattern recognition receptors (PRRs) by pathogen-associated molecular patterns (PAMPs) and damage-associated molecular patterns (DAMPs) activate microglia. These PAMPs come in the form of molecules derived from bacteria, viruses, and fungi; DAMPs typically form through leakage of intracellular contents, like nucleic acids, from necrotic cells. Inflammatory signals from nearby cells can also stimulate receptors on microglia, activating them into a neurotoxic phenotype. This binding initiates several intracellular signaling pathways that lead to downstream expression of neuroinflammatory molecules. Two pathways of interest are the nuclear factor kappa B (NF-kB) and mitogen-activated protein kinase (MAPK) signaling cascades (Doens and Fernández, 2014). Upon activation, microglia increase migration through the brain parenchyma and enhance their phagocytic capacity (Lively and Schlichter, 2013). Through these mechanisms, microglia protect the brain from pathogens and other neurotoxicants. Although necessary to protect the brain, continued activation of microglia can cause damage to neurons. In the healthy brain, microglia regulate their activity and function, with the help of astrocytes, allowing them to dynamically transform between activated and resting forms. In the aging brain, however, cellular dysfunction biases them towards neurotoxic phenotypes that promote prolonged neuroinflammation, which propagates CNS damage over time (Stojiljkovic et al., 2022).

## 3 Altered microglial function associated with age

In the aged brain, microglia have dystrophic morphology, reduced normal functioning, and increased susceptibility to senescence and pro-inflammation. Described as having increased soma volume, shortened process length, and fewer branched dendritic arbors, these cells demonstrate morphological differences compared to those in the healthy brain. Although commonly increased with age, dystrophic microglial cell numbers are also strongly associated with age-related disease states, including AD and Lewy body dementia (Shahidehpour et al., 2021). These morphological changes correlate with slower process motility, although increased somal movement is sometimes observed, which reduces overall immunological surveillance (Damani et al., 2011). As such, this contributes to the disproportionate populations of glia in the aged brain. In healthy tissue, microglia congregate in key regions, specifically the gray matter in murine models, and exhibit morphological and functional differences depending on the brain region they occupy (Lawson et al., 1990). In the aged brain, however, they are no longer as dispersed, instead residing in close proximity to one another within the tissue (Damani et al., 2011). Therefore, significant interference in surveillance and the reaction speed of aged microglia compared to young cells have been identified, making these cells less dynamic and slower to activate into fully functioning, reactive phenotypes (Hefendehl et al., 2014).

To compensate for this decrease in immune surveillance, microglial populations are often increased in the aged brain, but improper function makes this compensatory mechanism moot. As microglial populations increase with age, so do the proportion of dysmorphic cells (Shahidehpour et al., 2021). In addition to their inability to detect and react to ligands, their capacity to physically degrade them is also affected by age. Aged microglia improperly phagocytose and destroy, in part due to impaired production of degrading enzymes, including neprilysin and matrix metalloproteinase-9 (MMP9) (Suzanne et al., 2008). Upregulation of CD22, a receptor typically found on B cells, occurs with age and correlates to reduced phagocytosis in microglia (Pluvinage et al., 2019; Aires et al., 2021).

Age affects other aspects of the homeostatic functioning normally performed by microglia. They are involved in myelin remodeling and oligodendrocyte progenitor cell (OPC) differentiation, which is essential for axonal projections of neurons. Aged microglia actually suppress OPC differentiation, instead of promoting it as seen in the healthy brain (Luan W et al., 2021). Aged microglia can also push OPCs to form astrocytes, instead of mature oligodendrocytes, which prohibits myelination and therefore proper neuronal function. This adds to the cycle of pathogenesis perpetuated by glia, further contributing to a state of neuroinflammation in the brain that leads to neuronal degeneration (Baror et al., 2019). With age, myelin fragmentation increases over time, and microglia phagocytose this debris (Safaiyan et al., 2016). In addition to neuronal disturbance, myelin fragmentation overburdens microglial lysosomes, forming insoluble inclusions; this leads to microglial disfunction and senescence, contributing to the pathogenesis associated with age (Safaiyan et al., 2016).

These aged cells with altered function, or senescent microglia, display unique transcriptional profiles. Microglia in models of aging increase expression of pro-inflammatory cytokines, including tumor necrosis factor (TNF), interleukin-1β (IL-1β), and interleukin-6 (IL-6), even in their resting or inactive state (Sierra et al., 2007). One mechanism for prolonged inflammation is chronic upregulation of NF-κB signaling. Recent studies show that continued activation of this pathway can occur through leakage of lysosomal proteases by aged microglia, especially when the cells are overburdened, resulting in chronic neuroinflammation (Meng et al., 2020). When stimulated, aged microglia further enhance production of these pro-inflammatory molecules (Damani et al., 2011). Although slower reactivity is documented, these aged cells sustain baseline inflammatory signaling compared to healthy ones, resulting in excessive neuronal damage (Damani et al., 2011).

Importantly, glia can become more sensitive towards forming pro-inflammatory states. This priming can occur when the cells are repeatedly introduced to environmental stimuli, including infectious agents and neurotoxicants. This priming can be intensified by co-morbidities, disease, and age. The aging brain is characterized by a shift from homeostasis to an inflammatory state, with increased production of pro-inflammatory mediators and decreased anti-inflammatory ones. These conditions may also sensitize glia to produce an exaggerated response to the presence of an immune stimulus or other disturbance. It is hypothesized that this is caused by repeated stress weakening immunoregulatory mechanisms in glia, lowering the threshold required to form activated states. Stimuli can come in the form of overstimulation of TLRs by ligands encountered throughout the lifespan, such as lipopolysaccharide (Bukhbinder et al., 2022) and other bacterial toxicants, leading to glial tolerance and priming (Go et al., 2016). *Ex vivo* murine models show that when exposed to LPS, aged microglia are primed toward exacerbated production of the pro-inflammatory cytokines IL-1β and IL-6 and upregulate MHCII (Frank et al., 2010). This response is mediated by the CD200 receptor (CD200R) on microglia, which inhibits reactive cellular functions following stimulation. When exposed to stress, microglia downregulate CD200R, which interferes with the regulation of microglial responses to cellular stress, making them more prone to forming pro-inflammatory states (Frank et al., 2018). These exaggerated responses can have profound effects on the already delicate brain, resulting in permanent neuronal deficits that, eventually, lead to changes in cognition and motor function. Additionally, aged microglia increase the expression of various receptors, including scavenger receptors and TLRs (Cribbs et al., 2012). As such, aged animals are more sensitive to additional stimuli.

One mechanism for microglial dysmorphia, among the many that have been proposed, is altered intracellular iron homeostasis. Microglia express a protein, ferritin, that stores iron, a metal that accumulates in the aging brain. Increased iron accumulation and dysmorphic microglia are identified in aged non-human primates, although decreased ferratin^+^ microglial cells are found in the hippocampus and cortex. These findings suggest a pathological role for this imbalance in extracellular iron accumulation to microglial ferritin (Rodríguez-Callejas et al., 2019). Overall, accumulated iron results in oxidative stress and cellular damage (Kenkhuis et al., 2022).

## 4 Astrocytes are essential for neurological function and brain maintenance

The primary role of astrocytes is to provide support to neurons by stimulating them with growth factors, removing neurotransmitters from the synaptic cleft, and modulating ion and metabolite concentrations in the CNS. Importantly, astrocytes use changes in calcium concentrations, called calcium transients, to communicate with surrounding cells (Agarwal et al., 2017). Calcium transients result in neuron-glia crosstalk through neurotransmitter release, regulation of extracellular potassium concentrations, and vasoconstriction or vasodilation (Wang et al., 2012; Otsu et al., 2015). Altogether, these calcium transients contribute to learning and memory (Zhang et al., 2021).

Astrocytes also play a role in the development and maintenance of neuronal synapses. *In vitro* experiments show that astrocytes increase formation of synapses, in contrast to pure neuronal cultures (Pfrieger and Barres, 1997). This is likely from interactions with astrocyte-derived molecules, including thrombospondin and cholesterol, among numerous others (Mauch et al., 2001; Christopherson et al., 2005). In addition to synaptic plasticity, astrocytes support neurons through a glutamate-glutamine cycle, where the glial cells efficiently remove glutamate from the synaptic cleft. Glutamate is soaked up by astrocytes using the transporters GLT-1 and GLAST. This glutamate, through glutamine synthetase, is converted to glutamine and then supplied to neurons. Glutamine is essential for active neurotransmission by excitatory neurons, making astrocytes instrumental in not only neuronal signaling, but also preventing excitotoxicity and ultimately neuron death (Tani et al., 2014).

These cells also play a significant role in the formation of the blood-brain barrier (BBB), which acts to regulate cellular and biomolecular traffic into and out of the brain. Astrocytes physically contact the vasculature with the end of their processes, or “endfeet.” Transporters on these endfeet, like aquaporin-4 (AQP4), aid in molecular diffusion. Although vessel integrity is not entirely dependent on these endfeet, astrocytes produce proteins and growth factors, such as Wnt, that stimulate endothelial cell junctions and BBB tightness (Kubotera et al., 2019; Guérit et al., 2021).

When the brain is perturbed, astrocytes undergo morphological and functional changes that result in both neuroprotective or toxic functions. Although restricted compared to their glial counterpart, astrocytes constitutively express some TLRs and PRRs, in addition to cytokine and chemokine receptors (Carpentier et al., 2005; Jack et al., 2005). When these receptors are stimulated, astrocytes polarize from resting to reactive forms. Despite their association with the diseased brain, reactive astrocytes also demonstrate protective roles. Reducing astrocytic reactivity is correlated with increased disease severity in cases of neurodegeneration, like AD models. This is likely due to the phagocytic role astrocytes play in activated states; reactive astrocytes upregulate expression of MHC and aid microglia in the clearing of pathogens, debris, and protein aggregates (Vardjan et al., 2012; Morizawa et al., 2017). Though they perform protective roles, activated astrocytes also secrete pro-inflammatory mediators that, similar to microglia, can cause damage in the CNS over time.

## 5 Astrocyte function and age

The aging brain is characterized by astrocytes that reduce their normal function, while also maintaining a senescent, neuroinflammatory phenotype. Morphologically, astrocytes in the aged brain retain features of an activated cell. This includes cell body hypertrophy with increased complexity, such as number and length of their processes (Popov et al., 2021). Aging causes upregulation of glial fibrillary acidic protein (GFAP), which is typically associated with reactive phenotypes (Wu et al., 2005). Interestingly, astrocytic changes are region specific and not uniform across the entire brain. For example, cellular reactivity is found in the hypothalamus and hippocampus in particular (Suda et al., 2021). This regional diversity helps explain why these areas of the brain are more susceptible to damage and neurodegeneration.

Astrocytes from the aged brain upregulate pro-inflammatory cytokines, including IL-1β and TNF, and secrete proteins involved in the complement cascade (Boisvert et al., 2018; Suda et al., 2021). It is known that these complement proteins, especially complement component 1q (C1q), facilitate synapse engulfment by glia, which ultimately leads to synapse elimination and neuronal disfunction. Astrocytes are especially involved in the elimination of excitatory synapses, as proteins from this type of neuron are found in astrocytic lysosomes (Dejanovic et al., 2022).

Crosstalk is known to occur between astrocytes and microglia. Activated astrocytes, through their interleukin-10 receptors (IL-10R), secrete transforming growth factor beta (TGFβ) to attenuate microglial activation. With age, astrocytes both reduce IL-10R expression and become desensitized to IL-10, keeping them from secreting attenuating factors. This propagates pro-inflammation by both microglia and astrocytes (Norden et al., 2015; Norden et al., 2016). Altered attenuation by aged astrocytes occurs after treatment with LPS, demonstrating that this phenomenon ensues even following cellular stimulation (O'Neil et al., 2022).

Astrocytic calcium transients, which are involved in communication with the surrounding milieu, are altered with age. Aged astrocytes increase calcium transients in their primary branches but decrease transients in their endfeet (Ding et al., 2022). These fluctuations lead to improper potassium levels and impaired glutamate clearance, resulting in an excitotoxic and damaging environment for neurons (Popov et al., 2021). In addition to reduced transient numbers, decreased expression of aquaporin-4 (AQP4) occurs in the endfeet of aged astrocytes, which can affect water transport and ion concentrations in the brain (Ding et al., 2022). Not only are the proteins in endfeet unbalanced, but astrocytic contact with vessels changes with age as well. Although astrocytes in the healthy brain can replace their endfeet after ablation, this event is delayed in the aged brain (Mills et al., 2022). These irregularities in endfeet impact the integrity of BBB; astrocytes within the aged brain contribute to BBB dysfunction, which is commonly associated with age-related neurological diseases. This reduction in BBB function allows peripheral immune cells and toxic biomolecules to enter the brain, making the aged brain more susceptible to the effects of neurotoxicants (Pan et al., 2021).

## 6 Bacterial infections induce glial reactivity

Bacterial, especially intracranial, infections have been highly implicated in the formation of neurological deficits. The most common are meningitis-causing bacteria, which include *Escherichia coli*, *Neisseria meningitidis*, *Staphylococcus aureus, Streptococcus pneumoniae*, and *Mycobacterium tuberculosis* (Archibald and Quisling, 2013; Le Govic et al., 2022). These bacteria must first penetrate the BBB, the protective barrier that actively prevents microbes from entering the brain. Bacteria themselves can degrade the BBB to facilitate entry into the CNS. *Escherichia coli* (*E. coli*) interacts with toll-like receptor 2 (TLR2), and, through inflammation-induced upregulation of Vascular Endothelial Growth Factor A and Snail-1, activates the MAPK-ERK1/2 signaling cascade that modulates BBB proteins (Yang et al., 2016). This decreases expression of tight junction proteins, like occludin, which makes the barrier leaky. Similarly, *Neisseria meningitidis* disrupts junction proteins, through the help of matrix metalloproteinases, allowing for intercellular migration into the brain (Paul et al., 1998; Coureuil et al., 2009). Bacteria can also invade the brain without actually disturbing the BBB, as demonstrated by *Streptococcus pneumoniae* (*S. pneumoniae*) infection. Bacteria can enter the brain without disrupting endothelial cadherins, suggesting a paracellular route (Iovino et al., 2013). These mechanisms are some of the many methods bacteria use to degrade the BBB, allowing them to penetrate the brain and induce cellular stress.

Microglia play an essential role in the neuroinflammatory response induced by bacterial infections, due to the presence of their TLRs and PRRs. One PRR of interest is macrophage receptor with collagenous structure (MaRCo), which is constitutively expressed on microglia. Stimulating this receptor, as well as its coreceptor TLR2, activates the NF-κB pathway (Wang et al., 2021). This signaling cascade, through interactions between long non-coding RNAs and the interleukin-1 receptor-associated kinase 1 (IRAK1), facilitates pro-inflammation (Xu et al., 2022). In addition to NF-κB, MAPK signaling is triggered, further generating pro-inflammatory cytokines, including IL-6, interleukin-8 (IL-8), TNF, and IL-1β (Liu et al., 2018). Astrocytes are also recruited in the neurotoxic response associated with bacterial infections, although these mechanisms are not as well known. Intracerebral *S. pneumoniae* infection interferes with astrocytic glutamine synthetase activity in the hippocampus, leading to buildup of glutamate in the synapse and subsequent excitotoxicity (Tumani et al., 2000).

Although not as thoroughly investigated as those in the CNS, the effects of peripheral bacterial infections must not be overlooked. One way peripheral infections induce neurotoxicity is through bacterial components, which can reach the brain and cause persistent inflammation, even without physical dissemination of live bacteria to the CNS. Bacterial components include cell wall proteins like LPS, enzymes, toxins such as pertussis toxin (PTX), and bacterial DNA, which is highly immunogenic due to the high frequency of unmethylated cytosine-guanine (CpG) motifs. Shedding by both living and apoptotic bacteria releases these constituents into the bloodstream. Bacteria can also release extracellular vesicles containing LPS, nucleic acids, and proteins into the systemic circulation (Tulkens et al., 2020). Altogether, these bacterial PAMPs readily enter the brain, especially when the BBB is compromised, which is a common phenotype in aged mice. CpG exposure leads to glial reactivity, through toll-like receptor 9 (TLR9), and memory deficits in murine models, implicating the significance of peripheral bacterial infections and their potent impact on the brain (Dalpke et al., 2002; Tauber et al., 2009). Neuraminidase (NA), an enzyme found in some pathogenic bacteria, is an agonist for toll-like receptor 4 (TLR4), and to some extent TLR2, which directly cause microglia to activate and proliferate (Fernández-Arjona et al., 2019; León-Rodríguez et al., 2022). Intracerebroventricular administration of NA results in an enhanced glial response in the amygdala and hypothalamus in combination with anxiety-like behaviors (León-Rodríguez et al., 2022). LPS not only induces glial activation, but physically alters the BBB. *In vitro* studies of LPS exposure show changes in endothelial cells, reducing integrity of tight junction proteins. It also stimulates IL-1β production by microglia, which ultimately interferes with presynaptic proteins and disrupts neuronal signaling. Specifically, interleukin-33 (IL-33) is implicated in the neuroinflammatory response to LPS, as it is not only upregulated by *in vivo* models but activates cerebral endothelial cells, recruiting both immune cells and microglia (Cao et al., 2018). On a cellular level, microglia exposed to LPS dysregulate iron-related metabolic proteins, causing intracellular deposition of iron (Fernández-Mendívil et al., 2021). Lipoteichoic acid (LTA), another bacterial cell wall constituent, leads to a robust, *in vitro* inflammatory response by both microglia and neurons alike (Howe et al., 2020). Altogether, these studies demonstrate that, through glial reactivity, microbial components lead to the degradation of neurons (Sheppard et al., 2019). It is important to note that some microbial components are more immunogenic than others, and can vary between bacterial strains. For example, LPS generated from *E. coli* is a stronger TLR agonist than other LPS-generating strains, like *Porphyromonas gingivalis*, and produces a more pronounced pro-inflammatory response (Qiu et al., 2021).

Peripheral infection of multiple organ systems is implicated in neurotoxicity, including the gastrointestinal tract, urinary tract, and respiratory system. Chronic stomach infection by *Helicobacter pylori* is correlated with increased risk for neurodegenerative disease. In both *in vitro* and *in vivo* studies, outer membrane vesicles produced by *Helicobacter pylori* bacteria reach the brain, leading to astrocytic increases in GFAP and vimentin, activation of NF-κB, and the release of interferon γ (IFNγ). Such glial reactivity leads to inhibition of neurite outgrowth and neuronal damage (Palacios et al., 2023).

One of the most common causes of neuroinflammation from bacterial infections outside of the CNS are urinary tract infections (UTIs). These are often caused by infection with *E. coli,* as well as *Klebsiella pneumoniae, Proteus mirabilis, Enterococcus faecalis,* and *Staphylococcus saprophyticus* (Flores-Mireles et al., 2015). In one study, rats who received intra-urethral injections of *E. coli* demonstrated behavior changes. A deeper look into the brain showed elevated IL-1β in combination with reduced expression of growth factors, which ultimately prohibited proliferation of neural stem cells (NSCs) as early as 4 days post infection (dpi), and decreased neurogenesis at 34 dpi in the hippocampus (Darwish et al., 2022). Another study of UTIs in mice showed similar findings. Mice inoculated with *E. coli* demonstrated memory impairments, tested through the open field and Y-maze tests, and elevated levels of neuronal cleaved caspase-3 and IL-6 (Rashid et al., 2021).

The effects of peripheral infection are also seen in studies better understanding the gut-brain axis, where the microbiome induces neuroinflammation and worsens neurodegenerative disease. In murine PD models, in mouse strains that overexpress alpha synuclein, gut bacteria promote PD pathology, including motor deficits and microglial activation. These neurodegenerative effects were attenuated with antibiotic treatment (Sampson et al., 2016). One hypothesis for such neurotoxicity stems from the microbial components secreted into the blood, including LPS, enterotoxins, and small non-coding RNA (sncRNA).

In addition to the gut, a neurological connection is seen between the lung and the brain. Although *Mycobacterium tuberculosis* can disseminate to the CNS, neurological effects are seen without evidence of bacteria in the brain. In a guinea pig model of pulmonary disease, significant microglial proliferation, followed by an increase in astrocytes, is seen. Interestingly, a small, but insignificant, increase in the number of astrocytes is found soon after infection. This progressive glial reactivity preceded cognitive deficits, misfolded protein accumulation, and neuronal loss in the hippocampus (Latham et al., 2023). Pulmonary infection with another bacteria, *Pseudomonas aeruginosa*, decreased expression of the BBB proteins VE-cadherin and claudin-5, led to anxiety-like behaviors, and recruited microglia. Pro-inflammatory cytokines were also detected in the brain. Interestingly, these effects were found without evidence of bacteria in the CNS (Villalba et al., 2023).

The effects of bacterial infections can be exacerbated by a viral stressor. Co-infection with *S. pneumoniae* and influenza A virus in the lungs cause activated microglial morphology and the secretion of pro-inflammatory cytokines in the hypothalamus. This demonstrates that co-infection further exacerbates pathology compared to bacterial infection alone, as viruses induce their own neurological effects (Wang et al., 2018).

## 7 Viral infections, in the central nervous system and peripheral tissues, result in dramatic neuroinflammatory responses

Viral infections account for a high number of cases of encephalitis, or inflammation of the brain. Of these confirmed cases, the majority are the more vulnerable children and elderly populations. The most common neurotropic viral infections include herpes simplex virus (HSV), West Nile virus, and influenza A. Some of the others include varicella-zoster virus, Epstein-Barr virus (EBV), cytomegalovirus (CMV), herpes virus, eastern equine encephalitis virus (EEEV), western equine encephalitis virus (WEEV), zika virus, and dengue virus (Turtle and Solomon, 2012). These viral infections of the CNS are strongly associated with neurological disease because they induce pro-inflammatory glia that culminates in the degradation of neurons. Multiple neuroinflammatory pathways are shared between viral infection and neurodegenerative disease, especially Alzheimer’s Disease (AD). These include glial-mediated signals like MAPK, Rap1, NF-κB, and TNF (Li et al., 2022).

Microglia are typically the first to respond to CNS infection with a neurotropic virus. This is primarily due to viral ligands activating TLRs on microglia. Japanese Encephalitis virus (JEV) overexpresses miRNAs which act on toll-like receptor 7 (TLR7), resulting in NF-κB signaling followed by production of TNF and caspase activation that can damage neurons over time (Mukherjee et al., 2019). Expanding upon this, infection with Theiler’s murine encephalomyelitis virus (TMEV), which directly infects microglia, leads to the production of interferons, IL-1β, IL-6, interleukin-12 (IL-12), TNF, chemokine ligand 2 (CCL2), chemokine ligand 3 (CCL3), chemokine ligand 5 (CCL5), and inducible nitric oxide (iNOS). Secretion of these mediators by microglia has the potential to be sustained over prolonged periods of time (Olson et al., 2001). Interestingly, these microglial responses occur irrespective of the viral load, as even small inoculums result in highly ramified, activated microglia with increased ionized calcium-binding adaptor molecule 1 (Iba-1) expression (Howe et al., 2022). Exosomes secreted from *in vitro* TMEV-infected microglia can be engulfed by surrounding glia and neurons, which leads to subsequent production of pro-inflammatory molecules by those cells, such as interferon alpha (IFNα) and interferon beta (IFNβ), IL-6, IL-12, and TNF, and CCL2. This perpetuates inflammation throughout the brain. TMEV-infected microglia can also secrete viral RNA in these exosomes, which maintains infection over time, leading to chronic neuroinflammation (Luong and Olson, 2021).

Another encephalitic virus, the mosquito-borne alphavirus western equine encephalitis virus (WEEV), is implicated in the formation of parkinsonian pathology. The brains of outbred CD-1 mice infected with a non-lethal encephalitic dose of WEEV are characterized by the activation of glia followed by dopaminergic neuron loss (Bantle et al., 2019). Viral infections not only induce neuroinflammation, but directly affect the formation of neurotoxic α-synuclein aggregates characteristic of Parkinson’s Disease. Misfolding of α-synuclein into aggregated Lewy bodies, an event identified in the pathogenesis of PD, is exacerbated by injection with synthetic viral dsRNA. In this murine model, increased misfolding due to viral ribonucleic acid was followed by the degeneration of nigrostriatal neurons and PD-like motor deficits (Olsen et al., 2019).

Similarly, the consequences of infection with the flavivirus West Nile Virus (WNV) suggests microglial reactivity as a key player in the neurotoxicity seen in patients recovering from this neuroinvasive disease. In the study, mice infected by footpad inoculation of WNV demonstrate complement-mediated microglial activation that led to synaptic terminal loss, even after infection was resolved (Vasek et al., 2016). In a study using murine models of viral encephalopathies, microglial proliferation and transcriptional indications of activation were identified. These microglial changes were strongly associated with the progression of neuropathology, including neuronal apoptosis (Jhan et al., 2021). Zika virus encephalitis also involves microglial reactivity, as determined by morphological changes and increased phagocytic capacity (Enlow et al., 2021).

Interestingly, neurological effects can be observed after neurotropic viral infection has been attenuated, demonstrating prolonged neurotoxicity that is not in direct response to the virus. Patients infected with WNV have neurological deficits that persist even after the infection was cleared. This is likely due to a post-infectious pro-inflammatory brain state. In human WNV patients with persistent post-infectious symptoms, elevated levels of TNF is identified in sera (Leis et al., 2020).

Herpes simplex virus-1 (HSV-1) is a widespread neurotropic virus that can reach the brain, causing a rare form of encephalitis that can lead to long-term neurological deficits. In a murine model of herpes simplex encephalitis (HSE), via inoculation with attenuated virus, persistent microglial activation occurred. This was followed by neuronal loss and behavior deficits. These neuropathologies increase susceptibility to neurodegenerative disease, like AD (Wang et al., 2023).

Likewise, Human Immunodeficiency Virus (HIV) is strongly associated with neurological deficits, with approximately half of infected individuals facing CNS problems, called HIV-associated neurocognitive disorder (HAND). HIV readily enters the CNS, infecting microglia upon entry; brain regions involved in cognition and motor function, such as the prefrontal cortex, caudate nucleus, superior temporal sulcus, and the hippocampus, are especially vulnerable (Hawes et al., 2022). Neuroinflammation associated with this virus is mediated by microglial activation of the NLR family pyrin domain containing 3 (NLRP3) inflammasome and NF-kB, leading to elevated caspase-1 and IL-1β production by glia, both *in vitro* and *in vivo* (Chivero et al., 2017; Mamik et al., 2017; He et al., 2020). A similar study detected astrocyte proliferation with markers of oxidative stress (Byrnes et al., 2023). The glial response is not uniform across all brain regions, but is instead heightened in the midbrain compared to the frontal lobe, and contributes to overall synaptic dysfunction (Tavano et al., 2017; Garcia-Mesa et al., 2020). The neuroinflammation associated with HIV persists even when viraemia is suppressed and in response to attenuated virus. Analysis of brain tissue from macaques with suppressed infection or infection with attenuated Simian Immunodeficiency Virus (SIV) demonstrate microglial proliferation, activated astrocytes with increased GFAP, and upregulation of cyclooxygenase-1 and 2 (COX-1 and COX-2) that prolonged even after acute infection (Byrnes et al., 2023).

Viral infections of the brain have historically been implicated in neurodegenerative disease, especially parkinsonism. The first known infection being the influenza virus as long ago as 1918. Although not typically considered a neurotropic virus, influenza A can penetrate the brain, as seen in both humans and in animal models. *In vitro* studies show increased transcription of inflammatory cytokines, chemokines, and type I interferons by neurons upon infection in culture, but limited viral replication was seen in these cells (Wang et al., 2016). The limited replication in neurons, combined with neuroinflammatory molecule release, indicates a critical role of glia in the pathogenesis associated with influenza. In addition to neurons, both microglia and astrocytes can be infected with influenza virus in culture, although astrocytes do not generate infectious progeny, but lead to the expression of pro-inflammatory cytokines (Ng et al., 2018). Similarly, *in vitro* experiments show that when infected with the H5N1 strain of influenza, limited viral replication was seen in astrocytes despite significant production of IL-1β (Pringproa et al., 2018). Viral antigens from the influenza virus can activate TLRs on glia. Neuraminidase, a sialidase found on the influenza virus, is a ligand for toll-like receptor 4 (TLR4), and to some extent TLR2, on microglia. This activates the cells and induces an inflammatory reaction (Fernández-Arjona et al., 2019). Ultimately, acute influenza infection leads to spine loss in the hippocampus, reduced long-term potentiation in the hippocampus, and impairment in the formation of spatial memories. Interestingly, these long-term CNS alterations occurred in non-neurotropic strains of the virus as well (Hosseini et al., 2018).

This common pathogen has been repeatedly involved in long-term neuropathology, even during solely peripheral infection and after the infection has resolved. In both neurotropic and non-neurotropic strains of the virus, spine loss and reduced structural integrity of neurons is correlated with spatial memory loss. A potential mechanism for these neurodegenerative effects is the induced microglial reactivity in this region, with upregulation in the pro-inflammatory cytokines IL-1β, IL-6, TNF-α, and IFN-α, as well as reduced neurotrophic factors nerve growth factor (NGF) and brain-derived neurotrophic factor (BDNF) (Jurgens et al., 2012; Hosseini et al., 2018). This neuroinflammation in response to pulmonary influenza infection could be initiated by vagal innervation. In mice infected with influenza A, vagal sensory neurons upregulate expression of pro-inflammatory genes, inducing a neuronal inflammatory phenotype that leads to neuroinflammation (Verzele et al., 2021). Formalin-inactivated H7N7 vaccines reduce pro-inflammatory cytokine and chemokine levels, decreased microglial cell number, and induce increased branching complexity of these cells, preventing synapse loss in mice (Demuth et al., 2023).

Other viruses lead to both neurotropic and non-neurotropic infections. The consequences of the recent severe acute respiratory syndrome coronavirus 2 (SARS-CoV-2) pandemic has shed light on the CNS effects of peripheral viral infection. SARS-CoV-2 can cross the BBB and directly infect neurons and glia in the olfactory cortex and interconnected regions, inducing a neuroinflammatory response in both mice and non-human primate models (Beckman et al., 2022; Jeong et al., 2022; Erickson et al., 2023). Not only does glial reactivity occur as a result, producing proinflammatory cytokines including IL-1β, IL-6, and TNF while downregulating IL-10, but SARS-CoV-2 infection causes endoplasmic reticulum (ER) stress. This ER stress activated death receptors, promoting apoptosis of microglia (Jeong et al., 2022). Patients recovered from the viral infection experience cognitive deficiencies, which are likely explained by neuronal dysfunction and microglial reactivity. Both murine models and humans with mild SARS-CoV-2 respiratory infection demonstrate significant microglial proliferation in the white matter and upregulation of pro-inflammatory cytokines, even after the infection was resolved (Hampshire et al., 2021; Fernández-Castañeda et al., 2022).

## 8 Age, glia, and pathogens converge into a neurotoxic state

Over time, research has uncovered evidence of the neurological effects of infection with bacteria and viruses, both in the CNS and in peripheral tissues. A common theme exists where signaling molecules or PAMPs act as ligands for glial receptors. This pushes astrocytes and microglia into reactive, pro-inflammatory phenotypes that, eventually, irreversibly damage neurons. Considering this, it is easy to see how the effects of infectious agents can push the increasingly sensitive, aged brain into a state of disease. Especially when you take into account how susceptible to infection aging populations are, in combination with their sometimes extensive histories of infection.

Elderly patients are prone to being immunocompromised and malnourished, increasing their vulnerability to infectious agents. Vitamin D deficiency, which is often experienced in the aging population, correlates with increased levels of pro-inflammatory cytokines in an *E. coli* model of meningoencephalitis, highlighting its role in vulnerability to infection and neurotoxicity in the CNS (Djukic et al., 2015). The BBB also degrades with age, with reduced expression or function of proteins related to transport and tight junctions (Morita et al., 2005; Bartels et al., 2009; Sandoval and Witt, 2011). This allows for increased infiltration of immune cells, and potentially pathogens, into the aged brain (Pan et al., 2021).

Not only are the elderly more susceptible, but glial disfunction induced by age makes infections more severe. Experiments using intraperitoneal injection of *E. coli* in aged mice showed not only more microglia in the brain, but increased activation of these cells as determined by their morphology and pro-inflammatory cytokine secretion (Hoogland et al., 2021). Studies of peripheral *E. coli* infection show similar results, including long-term memory loss in aged, but not young, rats as well as increased levels of IL-1β (Barrientos et al., 2006).

In addition to being immunocompromised, elderly individuals likely display a medical history littered with numerous viral and bacterial infections. It is estimated, based on detectible antibodies in sera, that the average person has been exposed to 10 different viral species in their lifetime (Xu et al., 2015). This estimation does not account for additional infections by bacteria, or the effects of potential compounding factors that may place a person at increased risk of infection. Infection early in life actually exacerbates cognition decline in models of aging. In one experiment, rats exposed to *E. coli* shortly after birth had impaired memory and signs of gliosis at 16 months of age compared to uninfected animals (Bilbo, 2010). Furthermore, BBB disruption, which occurs during viral and bacterial infection, early in life may make the aging brain even more susceptible to neuronal damage. In a mouse model of AD, LPS treated mice had a leaky BBB (Barton et al., 2019). In a separate study of young and aged rats with induced BBB disfunction, aged animals demonstrate more severe neuronal damage and neurological deficits (DiNapoli et al., 2008). Knowing that repeated stress, including components of infectious agents, can prime glia to form exaggerated states of activation when stimulated, it is easy to conjecture that these primed cells may have a substantial effect on the aged brain.

Such a hypothesis is logical based on current investigations of primed cells. In a study using young and aged mice injected with LPS, aged mice displayed sickness behavior while the young animals did not. This was likely due to the enhanced production of pro-inflammatory cytokines that occurred in the aged animals (d’Avila et al., 2018; Huang et al., 2008). Glial priming has also been shown to result in dopaminergic neuron loss in the substantia nigra, a neuropathology associated with PD. Exposure to paraquat, a neurotoxic herbicide, causes cellular stress and neuronal loss in this region, although multiple exposures are necessary. Mice treated with LPS prior to exposure to a single dose of paraquat, however, lost dopaminergic neurons, highlighting the detrimental effects of primed glia (Purisai et al., 2007).

It is known that infectious agents play a role in disease, because they have been directly tied to neurodegeneration. The presence of lipopolysaccharide-binding protein, which is produced in response to LPS exposure, make individuals significantly more at risk for AD and dementia (André et al., 2019). Infectious agents have also been identified in the brains of post-mortem patients; *E coli*, LPS, and various other strains of bacteria have been found in human PD and AD brains, indicating that there could be a connection between infection and disease state (Zhan et al., 2016; Pisa et al., 2020; Emery et al., 2022). Along those lines, TLRs are upregulated in neurodegenerative disease. PD models using TLR4 knockout mice had reduced glial reactivity and neuroprotection of dopaminergic neurons (Paula et al., 2019). Protection from viral infection also reduces risk of neurodegenerative disease, as seen in cohort studies showing that people who received the Influenza vaccine have reduced risk of AD (Bukhbinder et al., 2022). Overall, these experiments indicate that infectious agents are involved in the neuropathology of disease, even if the specificities of their roles have not been elucidated.

This makes sense, as bacterial and viral toxicants have been implicated in the progression of neuropathology. Bacterial components, like bacterial DNA and LPS, colocalize with amyloid plaques in the AD brain, promoting aggregation of this toxic, misfolded protein (Zhan et al., 2016; Tetz and Tetz, 2021). *In vitro* exposure to the bacterium *Borrelia burgdorferi* and LPS increased expression of proteins involved in the pathogenesis of AD, including the amyloid β precursor protein (APP) and hyperphosphorylated tau (Miklossy et al., 2006). Expanding upon this, microbial sepsis augmented amyloid plaque load in AD mouse models (Basak et al., 2021). Not only do infectious agents affect the production of these proteins, but glial disfunction may make the brain unable to effectively clear neurotoxic proteins. Microglia in AD models are unable to properly phagocytose and degrade amyloid β fibrils, leading to the formation of insoluble aggregates (Wegiel et al., 2001; Suzanne et al., 2008).

It is also known that infection exacerbates the symptoms and neuropathology associated with neurodegenerative disease. Peripheral influenza A infection in an AD mouse model enhanced cognition deficits, glial reactivity, amyloid β plaque load, and neuronal degeneration compared to uninfected or wildtype animals (Hosseini et al., 2021). Pulmonary infection with *Bordetella pertussis* in an AD model caused helper T cells, secreting IFNγ and interleukin-17, and natural killer T cells to infiltrate the brain, activate glia, and stimulate amyloid β deposition (Browne et al., 2013; McManus et al., 2014). In another AD model, using a senescence-accelerated mouse-prone 8 (SAMP8) strain, intracerebroventricular SARS-VoV-2 infection showed increased glial activation compared to their CD-1 mouse counterpart (Erickson et al., 2023).

### 8.1 Glia perform conflicting roles within the brain

While substantial evidence describes neurotoxicity mediated by glia, their protective mechanisms must not be overlooked. Astrocytes can form physical barriers, or glial scars, that restrict cytosis after pathological events. They also secrete anti-inflammatory factors and regulate the microglial response. Through this glial crosstalk, microglia can regulate astrocytes and suppress inflammation. Activity such as debris clearing and promoting neurogenesis makes microglia critical in neuroprotective responses. As such, studies of glial depletion, utilizing both chronic and acute ablation, report that glia maintain neuronal integrity in the brain. Short-term microglial depletion results in a type 1 interferon inflammatory response, neuronal loss in the somatosensory cortex, and gait disturbances (Rubino et al., 2018).

Neuroprotective mechanisms by glia are apparent under pathologic conditions as well. Excitotoxicity induced by N-methyl-D-aspartic acid (NMDA) in an environment without microglia enhances neuronal death in the hippocampus, increasing the susceptibility of neurons to damage in anatomic regions that are usually less affected (Vinet et al., 2012). Selective depletion of proliferating microglia in a model of cerebral ischemia increased markers of inflammation and neuronal loss (Lalancette-Hébert et al., 2007). Ablating astrocytes after cortical injury also led to substantial neuronal loss and inflammation compared to control animals, although these results were only seen in mild to moderate injury (Myer et al., 2006).

Glia are instrumental in maintaining animal health in models of neurodegeneration as well, despite the role they play in the neurotoxicity associated with disease. In a model of PD, by murine exposure to 1-methyl-4-phenyl-1,2,3,6-tetrahydropyridine (MPTP), ablating microglia through inhibition of colony-stimulating factor 1 receptors (CSF1R) exacerbated parkinsonian symptoms and neuropathology. Mice lacking microglia had aggravated locomotor impairment and degeneration of dopaminergic neurons in the substantia nigra and striatum (Yang et al., 2018). Likewise, astrocytes and microglia are heavily involved in the progression of Alzheimer’s disease. Transgenic APP mice deficient in reactive astrocytes reduce clearing of monomeric amyloid β, had enhanced cognition deficits, and impaired neuronal synapses (Katsouri et al., 2020). Additionally, astrocytic ablation negatively affects neuronal plasticity, decreasing the number of dendritic spines (Davis et al., 2021). These trends apply to glial reactivity as well. Inhibiting the ability of astrocytes to activate, but not completely eliminating them, also exacerbates amyloid β deposition (Kraft et al., 2013). Microglia deficient in CC-chemokine ligand 2 (Ccr2), a chemokine receptor, in transgenic models of Alzheimer disease have an impaired glial response, leading to amyloid β accumulation and premature death of the organism (El Khoury et al., 2007). These protective mechanisms are due, partly, to the factors secreted by glial cells. This is shown in experiments of intraventricular treatment of glial cell line-derived neurotrophic factor (GDNF) in aged non-human primates. In the study, GDNF improved motor movement and dopamine release by neurons in the caudate nucleus, putamen, and substantia nigra (Grondin et al., 2003). The association between the beneficial activity of glia and pathological stressors, like the presence of cellular debris and misfolded proteins, potentially makes these protective mechanisms more pronounced in the aged brain.

## 9 Conclusion

With accumulating evidence that demonstrates alterations in glial behavior associated with age, it is still a mystery why some people progress into neurodegenerative disease, while others show signs of “healthy aging.” Accounting for the complexity of glial activity, by acknowledging that they function not only as pathological aggressors but also form protective roles, further complicates these scenarios. Viewing the brain as a complex “exposome,” or understanding that the culmination of exposures and environmental factors acquired over the course of a person’s lifetime impacts overall CNS health, helps explain why aging and disease are not straightforward from person to person. Considering the role infectious agents play on not only glia, but modulating neuroinflammation, proposes an important hypothesis for the progression of some brains, but not all, into states of disease and neurodegeneration.

**FIGURE 1:**
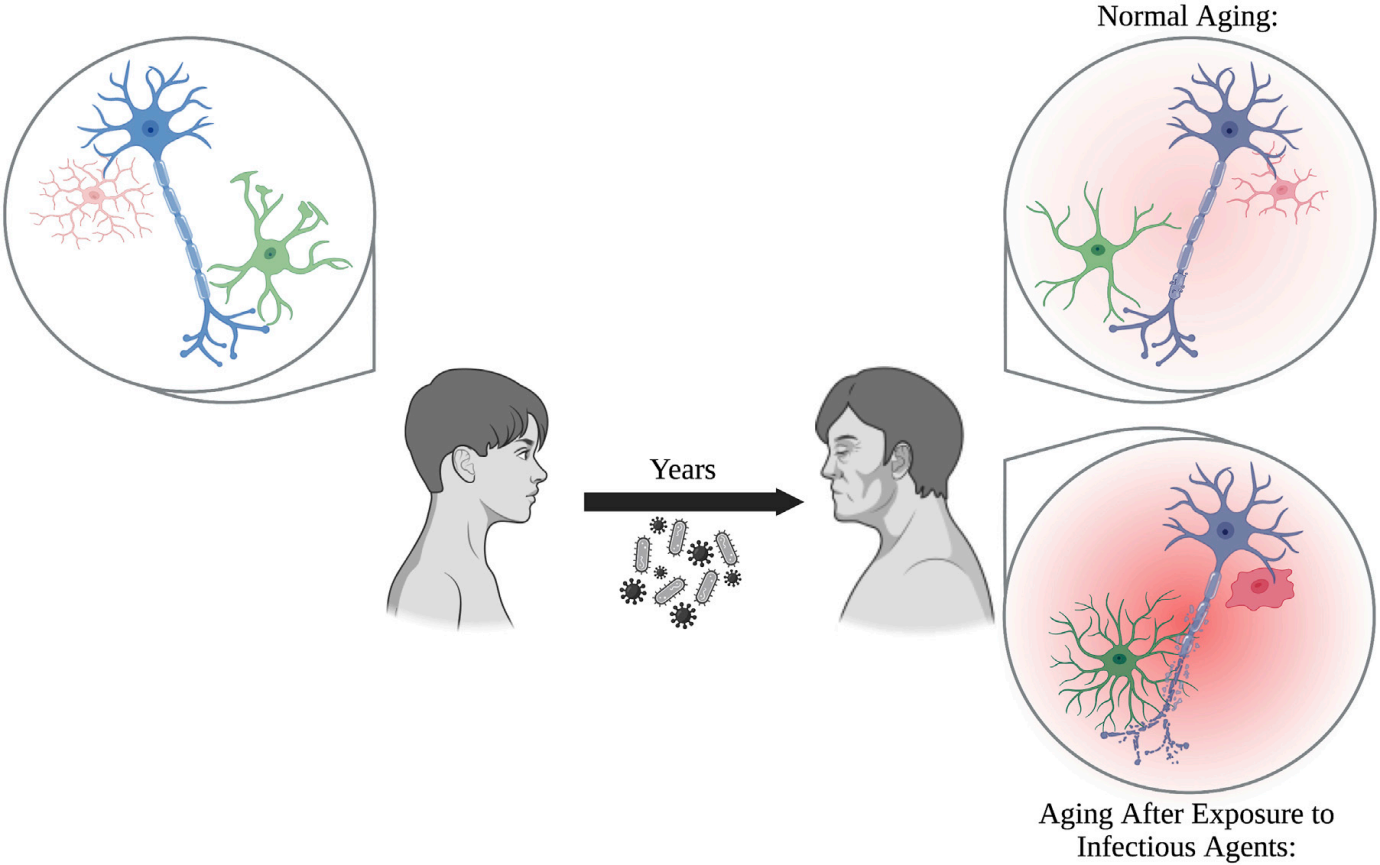
Glia perform critical functions by protecting the brain from neurotoxicants, maintaining homeostasis, and supporting neurons. During normal aging, glia become increasingly more reactive and senescent, which can be damaging to neurons over prolonged periods of time. Exposure to pathogens, including bacteria and viruses, over the course of a person’s lifetime prime glia into forming even more reactive, pro-inflammatory states. This results in an inflammatory brain phenotype that may exacerbate neuronal damage compared to normal aging alone.

With age, individuals are more susceptible to infectious agents, and often progress into more severe disease states such as sepsis. The aged brain, which is already more fragile due to low-level, sustained neuroinflammation mediated by glia, is prone to excessive damage if exposed to known stressors, including viral and bacterial infections (Figure 1). When you take into account the medical history accumulated over sixty + years, as seen in aged populations, it is possible that previous exposures may prime glia. This results in exaggerated responses when the balance within the brain is disrupted by additional stimuli, pushing the brain into neurodegenerative disease.
